# Multiple FNH-Like Lesions in a Patient with Chronic Budd-Chiari Syndrome: Gd-EOB-Enhanced MRI and BR1 CEUS Findings

**DOI:** 10.1155/2012/685486

**Published:** 2012-03-11

**Authors:** Caroline Newerla, Fabienne Schaeffer, Luigi Terracciano, Joachim Hohmann

**Affiliations:** ^1^Department of Radiology and Nuclear Medicine, University Hospital Basel, University of Basel, Petersgraben 4, 4031 Basel, Switzerland; ^2^Institute of Pathology, University Hospital Basel, University of Basel, Schönbeinstrasse 40, 4003 Basel, Switzerland

## Abstract

A-26-year old female patient with chronic Budd-Chiari syndrome due to different underlying blood disorders applied for a two-year followup of the liver with Gadolinium-ethoxybenzyl-diethylenetriaminepentaacetic-acid-(Gd-EOB-DTPA-) enhanced MRI. The liver function tests were raised. Besides showing a progressive hepatosplenomegaly and a cirrhotic liver alteration, the MRI revealed multiple new nodular lesions in all liver segments. These lesions showed typical patterns in the precontrast images, while there was an arterial and a persistent portal venous enhancement. In the hepatobiliary liver-specific late phase, a central “washout” and a persistent rim enhancement were observed (target sign). The additionally performed contrast-enhanced ultrasonography showed a strong zentrifugal arterial enhancement of the lesions followed by an isoechoic enhancement in the portal venous and delayed liver phase. Histologically these lesions turned out as focal nodular hyperplasias (FNH) or FNH-like lesions, also known as large regenerative nodules (LRNs). Differentiation between regenerative nodules like LRN and hepatocellular carcinoma (HCC) in cirrhotic livers is crucial, and the target sign in the hepatobiliary phase of Gd-EOB-DTPA as well as the centrifugal arterial enhancement followed by an isoenhancement during a CEUS might be useful for establishing the correct diagnosis of such hypervascular lesions with proliferated and likely aberrant bile ducts.

## 1. Introduction

The Budd-Chiari syndrome (BCS) is a rare vascular liver disease with a potentially severe course caused by drain disorder of the liver veins or of the inferior vena cava (IVC) resulting in portal hypertension. The etiology, the level of hepatic outflow tract obstruction, and the course of the disease differ between western and Asian countries [1]. Liver vein obstruction due to thrombosis predominates in western countries, whereas in China, Japan, and India, BCS is mainly caused by membranous obstruction of the IVC [2].

Therapeutic options include the positioning of shunts or IVC bypasses, the radical membrane resection with thrombus extraction, thrombolysis, angioplasty, stenting, and anticoagulation [3].

Progressed BCS may be associated with the development of liver cirrhosis and different focal liver nodes. In this context, the development of regenerative nodules which are resembling focal nodular hyperplasia (FNH) is likely [4] but has to be definitely differentiated from HCC nodules. 

## 2. Case Presentation

A-26-year-old female patient with chronic BCS known for three years applied for a two-year followup of the liver with magnetic resonance imaging (MRI). Further known diagnoses were a polycythaemia vera, a hereditary thrombophilia associated with a heterozygous factor V Leiden, a heterozygous factor VII deficiency, and a thalassemia. At the day of examination, the patient complained about abdominal discomfort lasting for weeks. Laboratory tests showed increased levels of bilirubin (27 *μ*mol/L, norm: 5–18 *μ*mol/L) and gamma glutamyl transpeptidase (GGT, 143 U/L, norm: 8–49 U/L) with otherwise normal transaminases, an increased alkaline phosphatase (ALP, 145 U/L, norm: 31–108 U/L), and an increased international normalized ratio (INR, 1.5, norm: INR < 1.3). 

The MRI was conducted on a 1.5 Tesla Avanto (Siemens, Erlangen, Germany) with a single body-array coil at the upper abdomen. Gd-EOB-DTPA (*Primovist*, Bayer Schering Pharma, Berlin, Germany) was applied as contrast medium via cubital aditus with a dosage of 0.1 mL/kg body weight and a flow rate of 2 mL/s. Gd-EOB-DTPA is a new gadolinium-based MRI contrast agent with a liver-specific hepatobiliary uptake and a liver-specific enhancement which starts about 10 min p.i. Subsequent to the precontrast sequences (T2-weighted HASTE, axial and coronal plane; T2-weighted TSE, T1-weighted FLASH 2D and VIBE with fat saturation (FS), axial plane), a dynamic T1-weighted examination (VIBE FS, axial plane; arterial: 30 s p.i., portal venous: 60 s p.i.) was carried out. The late hepatobiliary phase after 20 min p.i. (T1-weighted Flash 2D FS, axial and coronal plane, 20 min p.i.) completed the examination.

The CEUS was done on an Acuson Sequoia (Siemens, Mountain View, CA, USA) using a 4.2 MHz convex scanner in a contrast-pulse-sequency- (CPS-) mode (mechanical index, MI = 0.21). A sulphur-hexafluoride-based contrast agent (BR1, *SonoVue*, Bracco, Milano, Italy) was applied again over a cubital access, this time manually with a subsequent NaCl-Bolus of 10 mL. After the conventional B-mode imaging including a Doppler examination, the dynamic ultrasound was carried out over a time period of 5 min. The arterial phase, the portal venous phase, and the liver late phase (>2 min p.i.) were documented.

In the two-year interval, a progressive hepatosplenomegaly (craniocaudal extention of 20 cm versus 16 cm in the preliminary investigation) appeared as well as a cirrhotic liver alteration. Liver parenchyma showed an inhomogeneous perfusion pattern in the dynamic MRI sequences and pooling of contrast medium in the more central areas surrounding the porta hepatis during the late phase. Newly developed intrahepatic collaterals were also found and best visualized in the portal venous phase. Additionally multiple, also newly developed, nodular lesions appeared in all liver segments, the largest with a diameter of about 2 cm in segment VIII.

**Figure 1:**
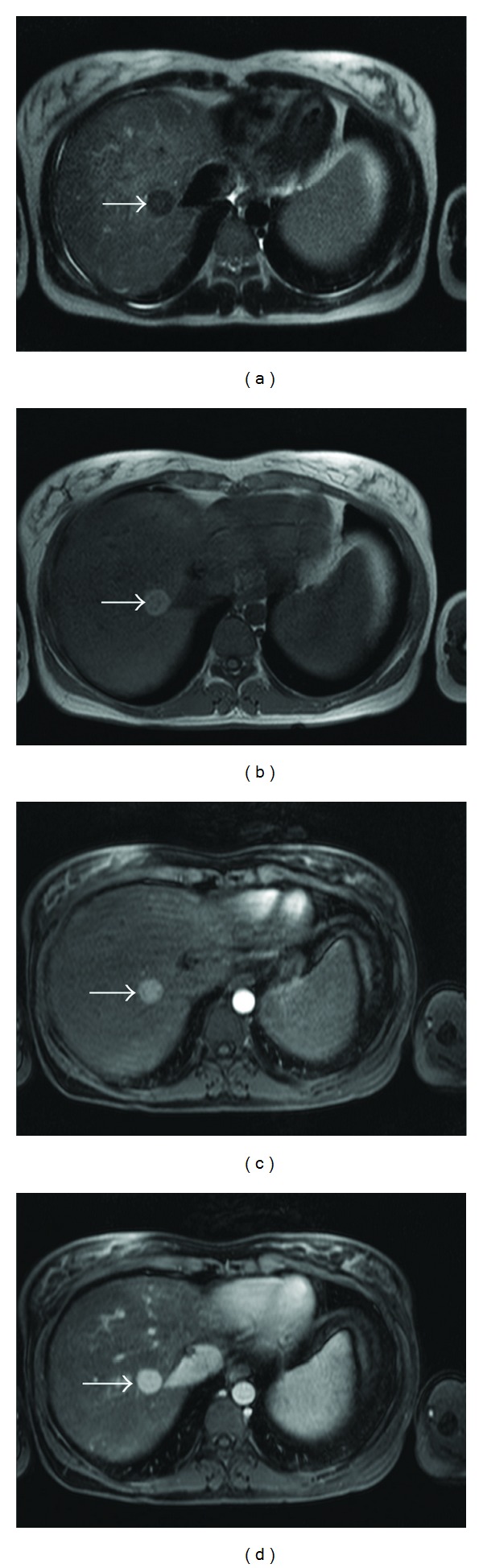
In T2-weighted HASTE sequence, (a) the reference lesion reveals hypointense (arrow). In precontrast T1-weighted FLASH 2D sequence, it is inhomogeneous hyperintense (arrow) (b). Arterial phase (c) and portal venous phase (d) T1-weighted VIBE show a progredient enhancement (arrows).

**Figure 2:**
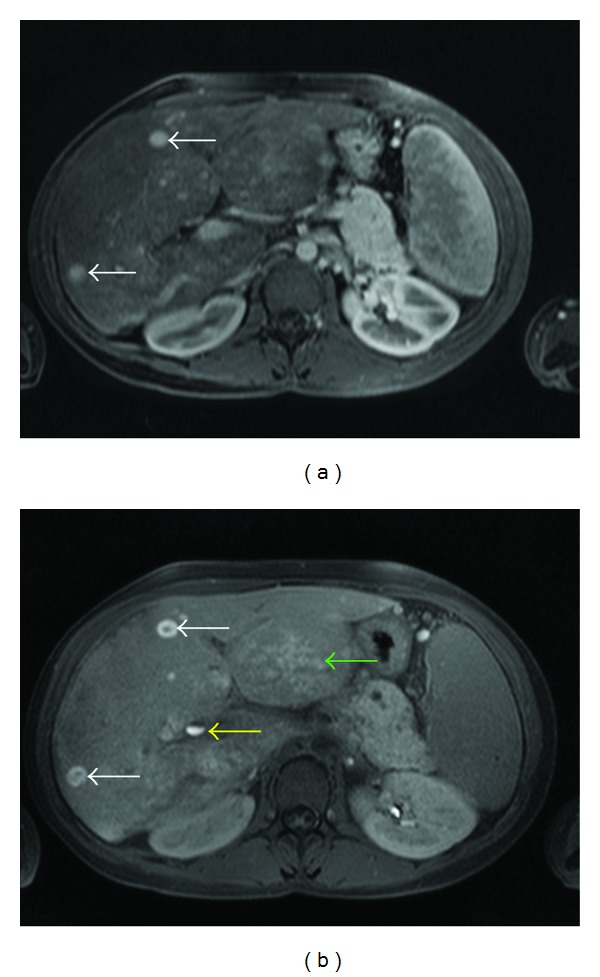
As the multiple lesions show a homogeneous enhancement in portal venous phase (a) (T1-weighted VIBE sequence), there is a central “washout” with a peripheral pooling of contrast agent (white arrow) in late phase (b) (T1-weighted FLASH sequence, 20 min p.i.). This is likely to be due to a missing washin of the more central parts. Additionally, there is an evidence of an inhomogeneous hilar pooling of the contrast agent, predominantly in the left liver lobe (green arrow). Contrast agent level in the ductus hepatocholedochus (yellow arrow).

These lesions were hypointense on T2-weighted images and inhomogeneous hyperintense on precontrast T1-weighted sequences. On dynamic postcontrast examination, all lesions showed arterial enhancement which persisted in portal venous phase (Figure 1). In the hepatobiliary liver-specific late phase, a central “washout” and a persistent rim enhancement (target sign), at least in larger lesions, was observed (Figure 2).

**Figure 3:**
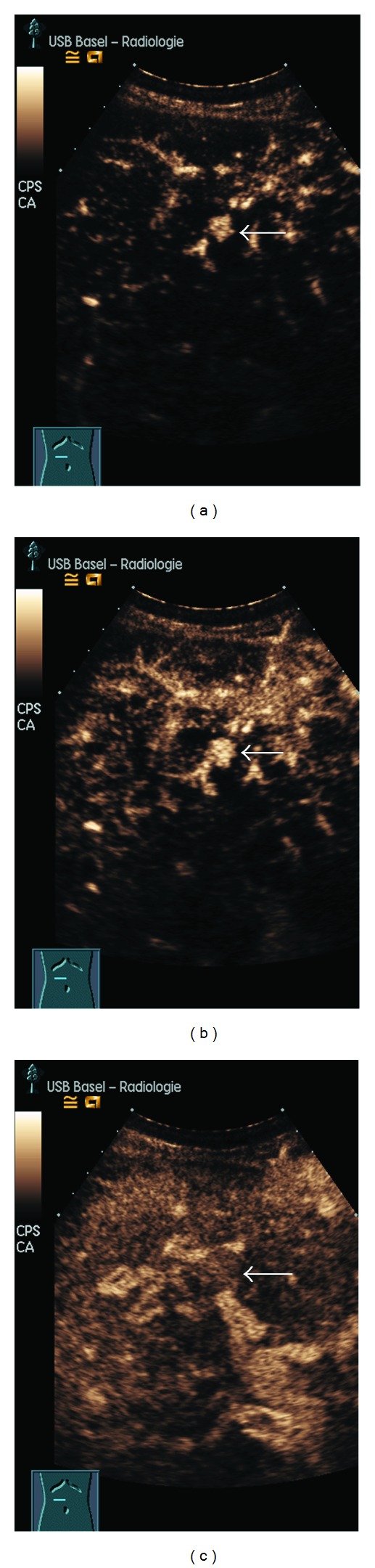
CEUS shows a more explicit centrifugal arterial (a) 16 s p.i.; (b) (17 s p.i.) and a liver like portal venous enhancement (c) (50 s p.i.) of the reference lesion (arrow).

CEUS showed, more distinctively than MRI, a strong arterial enhancement, which started in the center of the lesions and propagated to the peripheral parts. During the portal venous and the delayed liver phase, the lesions then appeared isoechoic compared with the surrounding liver parenchyma (Figure 3).

**Figure 4:**
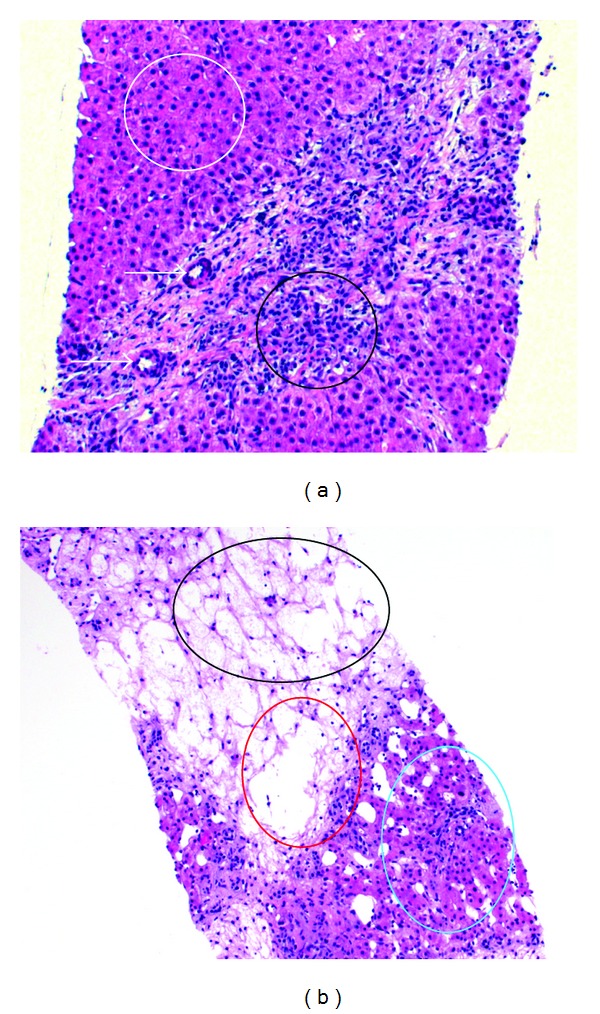
Histological preparation: (a) thickened arterial vascular wall (arrows), proliferation of bile ducts and of inflammatory cells (black), and normal hepatocytes/liver parenchyma (white) (b) dropout of hepatocytes as a sign of cirrhosis of the liver, liver vein (red), and normal liver parenchyma (light blue).

An ultrasound-guided biopsy of one of the lesions in segment VIII revealed an FNH or in the clinical context and together with the image findings likely an FNH-like lesion, also known as a large regenerative nodule (LRN) [5], and a cirrhotic alteration of the surrounding liver parenchyma (Figure 4).

## 3. Discussion

The development of regenerative nodules with FNH aspect in chronic BCS is a known condition. It is assumed that the impaired portal perfusion in chronic BCS is compensated by a progressive enlargement of the hepatic artery to maintain a steady hepatic inflow. This vascular imbalance with an increase of arterial perfusion in liver parenchyma is expected to support the development of large regenerative nodules, the arterial arborisation, and the development of aberrant bile ducts in these nodules being responsible for a histological FNH aspect [4, 5]. However, there are mainly two different types of nodular lesions that have been described with the history of impaired liver circulation: nodular regenerative hyperplasia (NRH) and large regenerative nodules (LRNs), the latter are the more FNH-like lesions [5, 6]. In general, these nodules develop independently from liver cirrhosis, and LRNs seem to be associated with BCS [5].

MRI appearance of the BCS depends on duration and extent of the obstruction as well as on portal venous flux changes. The acute stage shows congestion and hepatomegaly followed by a mild atrophy of liver cells; intrahepatic and subcapsular collaterals emerge. Chronic BCS turns into liver cirrhosis with focal or generalized nodular regrouping of liver parenchyma [7].

FNH-like lesions within a chronic BCS have been described in literature as small hypervascular lesions. Lesions bigger than 1 cm often show a central scar [8].

In this case, dynamic MRI and CEUS showed typical features of an FNH or an FNH-like lesion in terms of a strong arterial enhancement, followed by a contrast agent pooling in portal venous phase (Figures 1 and 3). MRI with GD-EOB-DTPA then showed a central “washout” of the lesions in the hepatobiliary liver-specific late phase while the contrast agent retained in the more peripheral part of the lesions (*target-sign*, Figure 2). Although we use the term “wash-out”, this is likely to be a missing washin or a missing pooling of the lesion parts which did not have proliferated aberrant bile ducts. This observed target sign in the hepatobiliary phase of Gd-EOB-DTPA might be useful for the characterisation of such lesions.

If we consider these FNH-like lesions as LRN, the precontrast T1- and T2-weighted sequences were rather typical (Figure 1). LRNs are usually hyperintense compared to the surrounding liver parenchyma in T1-weighted images and iso- to slightly hypointens in T2-weighted images [9–11]. This is different compared to regular FNHs which are usually slightly hypointense in T1 and slightly hyperintense in T2 [12].

Here, the lesions were hyperintense with merely central hypointensity on precontrast T1-weighted sequences (FLASH 2D, VIBE) and hypointense on the T2-weighted sequences so as to assume LRN [13]. The lesions are hyperintense on T1 because of a higher load of copper, while the hypointense appearance in T2-weighted images is likely due to the more regenerative character of the lesions compared with usual FNH.

Contrast-enhanced MRI (Figures 1 and 2) and CEUS (Figure 3) may therefore show the typical features of an LRN together with typical FNH pattern. In this case, an arterial enhancement was typical together with a venous pooling while a feeding artery, a spokewheel pattern, or a central scar could not be recognised. In addition, we found the described target sign on MRI and a clear visible centrifugal enhancement on CEUS.

LRN with FNH aspects in BCS are generally stable. Biopsy seems to be necessary only for overall uncharacteristic findings. Nevertheless, liver cell adenoma and highly differentiated hepatocellular carcinoma should be considered as differential diagnoses. Therefore, continuous controls combined with regular serum alpha-fetoprotein analysis are required.
